# Comparison of the clinical effectiveness of zanamivir and laninamivir octanoate for children with influenza A(H3N2) and B in the 2011–2012 season

**DOI:** 10.1111/irv.12147

**Published:** 2013-08-19

**Authors:** Naoko Koseki, Miki Kaiho, Hideaki Kikuta, Koji Oba, Takehiro Togashi, Tadashi Ariga, Nobuhisa Ishiguro

**Affiliations:** a Department of Pediatrics, Hokkaido University Graduate School of MedicineSapporo, Japan; b Pediatric Clinic, Touei HospitalSapporo, Japan; c Translational Research and Clinical Trial Center, Hokkaido University HospitalSapporo, Japan; d Sapporo City University School of NursingSapporo, Japan

**Keywords:** Biphasic fever, influenza, laninamivir octanoate, neuraminidase inhibitors, zanamivir

## Abstract

**Objectives:**

To evaluate the clinical effectiveness of the two inhaled neuraminidase inhibitors (NAIs), zanamivir (ZN) and laninamivir octate (LO), for influenza A(H3N2) and B virus infections.

**Design:**

A prospective, multicenter observational study was conducted from January to April in 2012.

**Setting:**

Outpatients aged 5–18 years who had a temperature of 37.5°C or higher and were diagnosed as having influenza based on an immunochromatographic assay were enrolled.

**Sample:**

A total of 338 patients treated with ZN and 314 patients treated with LO were compared.

**Main outcome measures:**

The duration of fever after administration of the first dose of each NAI was evaluated as a primary endpoint. The secondary endpoint was episodes of biphasic fever.

**Results:**

No statistically significant difference in the duration of fever was found between the ZN and LO groups (log-rank test, *P* = 0.117). A logistic regression model showed that episodes of biphasic fever increased by 1.19 times for every decrease of 1 year of age (*P* = 0.016) and that the number of biphasic fever episodes in patients treated with LO was 5.80-times greater than that in patients treated with ZN (*P* < 0.001).

**Conclusions:**

Although the duration of fever in the LO group was comparable to that in the ZN group, episodes of biphasic fever were more frequent in younger children and in the LO group than in the ZN group.

## Introduction

Oseltamivir (OT) and zanamivir (ZN) are neuraminidase inhibitors (NAIs) and are used worldwide for treatment and prophylaxis of influenza caused by influenza A and B viruses.^1^ Neuraminidase inhibitors can alleviate the major symptoms (fever, headache, myalgia, cough, headache, sore throat, etc.) of uncomplicated influenza A and B and reduce the duration by approximately 1–1·5 days when administered within 48 hours of onset of illness compared with a placebo.^2^–^4^ Neuraminidase inhibitors reduce the incidence of acute otitis media in children aged 1–5 years who are suffering from seasonal influenza^5^ and contribute to survival benefit in patients infected with influenza A(H1N1)pdm09 virus.^6^ In 2010, a long-acting NAI, laninamivir octanoate (LO), was approved for treatment of influenza A and B in Japan. Oseltamivir is administered orally, whereas ZN and LO are administered by oral inhalation. There was no significant difference in the time to alleviation of influenza A(H3N2) or B virus infection between patients treated with LO and OT.^7^,^8^ Comparisons of the clinical effectiveness of ZN and LO have been limited.^9^,^10^ In this study, we evaluated the clinical effectiveness of the two inhaled NAIs (ZN and LO) for influenza A(H3N2) and B virus infections by comparing the durations of fever after administration of the first doses of NAIs.

## Patients and methods

### Study design

A prospective, multicenter observational study was conducted from January to April in 2012 at 18 pediatric clinics and in department of pediatrics in 12 hospitals in Hokkaido, Japan. Outpatients aged 5–18 years who had an axillary temperature of 37·5°C or higher were diagnosed as having influenza virus infection based on results obtained by an immunochromatographic assay described below. Patients were excluded from this study if the time from onset of fever to initiating administration was 48 hours or more or if body temperature fell to <37·5°C before starting administration.

Patients diagnosed with influenza virus infection were assigned to receive either ZN or LO after obtaining informed consent from the children's parents. The decision regarding administration of ZN or LO was left to the discretion of the physician. Physicians prescribe anti-influenza drugs to almost all influenza patients in Japan regardless of the severity of influenza. Therefore, the bias in choice of ZN or LO by severity of influenza is minimal, if any. Zanamivir inhalation was performed twice daily for 5 days (20 mg per day), and LO was administered as a single inhalation (20 mg for patients <10 years of age and 40 mg for patients more than 10 years of age).

The age and sex of each patient and the date and results of the rapid diagnostic test were recorded by physicians. The time of onset (the first time that the patient had a fever of more than 37·5°C), vaccination status, selection of NAIs, date and time of first administration of NAIs, and total number of administrations of NAIs were recorded by the parents of children. The parents were also instructed to take their children's axillary body temperatures at least four times daily and to plot the body temperatures on a graph with temperature on the vertical axis and time on the horizontal axis. The time at which a temperature of <37·5°C was attained and maintained for more than 48 hours was defined as the time when the patient became afebrile. If a patient's temperature decreased to <37·5°C and remained <37·5°C for more than 24 hours but later increased to more than 37·5°C, the patient was considered to have biphasic fever.

### Immunochromatographic assay

Diagnosis for influenza virus infection was carried out with Clearline® Influenza A/B/(H1N1)2009 (Alere Medical Co., Japan), which can identify three types of influenza virus infections: influenza A(H3N2), influenza B and influenza A(H1N1)pdm09. The rest of the extraction buffer was kept in a −20°C freezer for further analysis.

### Real-time reverse transcription PCR

Real-time reverse transcription PCR was performed according to the referenced protocol.^11^,^12^

### Sample size and power calculation

Kawai *et al*.^13^ reported that the proportions of patients afebrile at 24 hours after the first dose of NAIs were 50% for patients with influenza A and 45% for those with influenza B in the ZN group. If 15% of the patients taking LO were afebrile within 24 hours, 80% power could be obtained if we performed the log-rank test with a two-sided 5% alpha with 640 patients in total based on nQuery Advisor (Statistical Solutions Ltd., Saugus, MA, USA).

### Statistical analysis

Demographic data are expressed as means ± SD or proportions. Variables related to time are shown as medians. We compared continuous variables using Student's *t*-test. Frequency analysis was performed by the χ^2^ test. The distributions of fever duration were depicted by the Kaplan–Meier method, and the log-rank test was used for comparisons of estimated fever duration between the ZN and LO groups. To adjust for confounding, we set the duration of fever as a dependent variable and set the following factors as clinically relevant independent variables in the multivariate Cox's regression analysis: type of treatment (ZN, LO), age, sex, vaccination status, and time from onset to first dose of the NAI. The logistic regression model was used to determine factors (age, sex, anti-influenza drugs, types of influenza, vaccination, and time from onset to inhalation) influencing the episodes of biphasic fever. A two-sided *P* value of *<*0·05 was considered statistically significant. All statistical analyses were performed using jmp software version 10.0.0 (SAS Institute, Cary, NC, USA).

## Results

### Patient characteristics

A total of 785 patients, who were otherwise healthy, were enrolled in this study. Patients who did not meet the criteria for this study, patients for whom complete clinical information was not available, and patients who had bacterial infections (pneumonia, otitis media) were excluded. A total of 447 patients were infected with influenza A(H3N2), and 205 patients were infected with influenza B. Two patients infected with A(H1N1)pdm09 were excluded due to the small number of patients. The demographic characteristics of the patients are shown in Table 1. Of the 338 patients treated with ZN, 234 were infected with influenza A(H3N2) and 104 were infected with influenza B. Of the 314 patients treated with LO, 213 were infected with influenza A(H3N2) and 101 were infected with influenza B. No significant differences were found between the ZN and LO groups in baseline status items: age, sex, vaccinations status, and time from onset of influenza virus infection to administration of NAIs. The numbers (doses) of ZN administration were five times (50 mg) in 4 patients, six times (60 mg) in 11 patients, seven times (70 mg) in seven patients, eight times (80 mg) in 10 patients, nine times (90 mg) in five patients, and ten times (100 mg) in 301 patients. No adverse reactions were reported in patients treated with ZN or LO.

**Table 1:** Background characteristics of patients infected with influenza A and B viruses

	Zanamivir	Laninamivir octanoate	*P*
No. of patients	338	314	
Age (year)			
Mean ± SD	9·4 ± 2·7	9·8 ± 2·7	0·070
Range	5–18	5–16	
No. (%) females	165 (48·8%)	140 (44·6%)	0·279
No. (%) males	173 (51·2%)	174 (55·4%)	
Vaccinated against influenza			
No. (%) vaccinated	176 (52·1%)	167 (53·2%)	0·776
No (%) not vaccinated	162 (47·9%)	147 (46·8%)	
No. (%) positive by rapid diagnostic test	338 (100%)	314 (100%)	
No. (%) of A(H3N2)	234 (69·2%)	213 (67·8%)	0·701
No. (%) of B	104 (30·8%)	101 (32·2%)	
Mean duration (hour) of illness before treatment ± SD	18·6 ± 11·5	20·1 ± 9·9	0·078

Of the 447 influenza A(H3N2)-positive and 205 B-positive samples by the rapid immunochromatographic assay, 100 and 50 samples were tested by real-time reverse transcription PCR, respectively. Influenza A(H3N2) viruses were confirmed to be H3 by PCR, while the B viruses were confirmed to be influenza B.

### Duration of fever after administration of the first dose of the NAI

**Figure 1:**
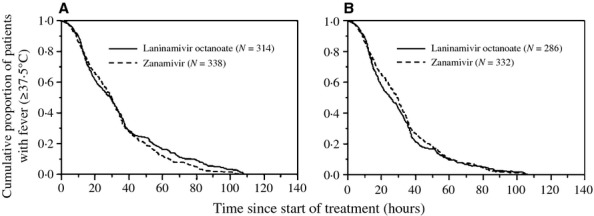
Kaplan–Meier curves showing a comparison of times taken for body temperature to return to <37·5°C in (A) zanamivir (ZN)- and laninamivir octanoate (LO)-treated patients (log-rank test: χ^2^ = 2·5, d.f. = 1, *P* = 0·117) and in (B) ZN- and LO-treated patients who did not have biphasic fever (log-rank test: χ^2^ = 0·403, d.f. = 1, *P* = 0·526).

The duration of fever after administration of the first dose of the NAI in the patients treated with ZN and LO was evaluated by Kaplan–Meier estimates (Figure 1A). No statistically significant difference in the duration of fever was found between the ZN and LO groups (log-rank test, *P* = 0·117). The median times were 29·5 and 28·8 hours for the ZN and LO groups, respectively.

**Figure 2:**
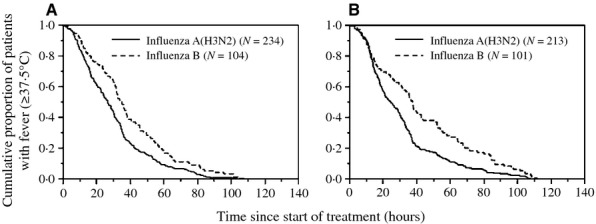
Kaplan–Meier curves showing a comparison of times taken for body temperature to return to <37·5°C in patients with influenza A(H3N2) and B who were treated with (A) zanamivir (log-rank test: χ^2^ = 10·5, d.f. = 1, *P* = 0·001) and with (B) laninamivir octanoate (log-rank test: χ^2^ = 15·2, d.f. = 1, *P* < 0·001).

Kaplan–Meier estimates for duration of fever after administration of the first dose of the NAI were generated to compare influenza A(H3N2)-infected and influenza B-infected patients (Figure 2). Log-rank tests demonstrated a statistically significant difference in duration of fever after administration of the first dose of the NAI between influenza A(H3N2)-infected and influenza B-infected patients (*P* = 0·001 for ZN and *P* < 0·001 for LO). The median times were 26·1 and 34·4 hours for influenza A(H3N2)-infected and influenza B-infected patients treated with ZN, respectively. The median times were 24·5 and 37·0 hours for influenza A(H3N2)-infected and influenza B-infected patients treated with LO, respectively.

**Figure 3:**
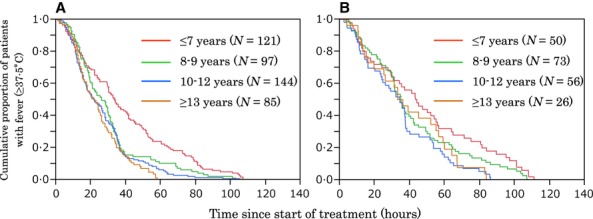
Kaplan–Meier curves showing a comparison of times taken for body temperature to return to <37·5°C in different age groups of (A) influenza A(H3N2)-infected patients (log-rank test: χ^2^ = 33·1, d.f. = 3, *P* < 0·001) and (B) influenza B-infected patients (log-rank test: χ^2^ = 9·6, d.f. = 3, *P* = 0·022).

The Kaplan–Meier estimates for duration of fever after administration of the first dose of the NAI were stratified by age groups (Figure 3). Log-rank tests demonstrated a statistically significant difference in duration of fever after administration of the first dose of the NAI between age groups (*P* < 0·001 for influenza A(H3N2) and *P* = 0·022 for influenza B). The median times were 33·5, 26·1, 21·7, and 21·9 hours for influenza A(H3N2)-infected patients ≤7, 8–9, 10–12, and ≥13 years of age, respectively. The median times were 43·7, 35·0, 34·2, and 34·5 hours for influenza B-infected patients ≤7, 8–9, 10–12, and ≥13 years of age, respectively.

The Cox's proportional hazards model showed that the duration of fever after administration of the first dose of the NAI was shorter in older patients (hazard ratio = 0·91 per 1 year of age, 95% confidence intervals of 0·88–0·93, *P* < 0·001) and that the duration of fever after administration of the first dose of the NAI was longer in patients with influenza B infection than in patients with influenza A(H3N2) infection (hazard ratio = 1·60, 95% confidence intervals of 1·35–1·90, *P* < 0·001) (Table 2). The duration of fever after administration of the first dose of the NAI weakly correlated with sex (*P* = 0·049) and time from onset to inhalation (*P* = 0·042). There was no statistically significant association between duration of fever following commencement of treatment and treatment regimen (*P* = 0·082) or vaccination status (*P* = 0·375).

**Table 2:** Results of Cox's proportional hazards model to determine factors influencing duration of fever after administration of the first dose of the neuraminidase inhibitor

Independent factors	Hazard ratio (95% Confidence interval)	*P* value
Age*	0·91 (0·88–0·93)	<0·001
Sex**	1·17 (1·00–1·37)	0·049
Anti-influenza drugs	1·15 (0·98–1·34)	0·082
Types of influenza***	1·60 (1·35–1·90)	<0·001
Vaccination	0·93 (0·79–1·09)	0·375
Time from onset to inhalation†	0·99 (0·99–1·0)	0·042

* The duration of fever after administration of the first dose of the NAI was shorter in older patients (hazard ratio = 0·91 per 1 year of age, 95% confidence intervals of 0·88–0·94, *P* < 0·001).

** The duration of fever after administration of the first dose of the NAI was longer in male patients than in female patients (hazard ratio = 1·17, 95% confidence intervals of 1·00–1·37, *P* = 0·049).

*** The duration of fever after administration of the first dose of the NAI was longer in patients with influenza B infection than in patients with influenza A(H3N2) infection (hazard ratio = 1·60, 95% confidence intervals of 1·35–1·90, *P* < 0·001).

† The duration of fever after administration of the first dose of the NAI was shorter in patients whose time from onset of influenza virus infection to administration of the NAI was longer (hazard ratio = 0·99 per 1 hour, 95% confidence intervals of 0·98–1·00, *P* = 0·042).

### Episodes of biphasic fever

Biphasic fever was observed in 19 (4·3%) of the 447 influenza A(H3N2)-infected patients and in 15 (7·3%) of the 205 influenza B-infected patients, totally in 34 (5·2%) of the 652 patients (Table 3). These patients did not have infectious complications such as pneumonia or acute otitis media. The frequency of biphasic fever was significantly higher in influenza-infected patients treated with LO than in patients treated with ZN: 28 (8·9%) of the 314 patients treated with LO and 6 (1·8%) of the 338 patients treated with ZN (χ^2^ test, *P* < 0·001) (Table 3A). When the patients were subdivided into influenza A(H3N2)- and B-infected patients, the frequencies of biphasic fever in both groups were significantly higher in patients treated with LO than in patients treated with ZN: biphasic fever occurred in 15 (7·0%) of the 213 influenza A(H3N2)-infected patients treated with LO and 4 (1·7%) of the 234 influenza A(H3N2)-infected patients treated with ZN (χ^2^ test, *P* = 0·005) (Table 3B) and in 13 (12·9%) of the 101 influenza B-infected patients treated with LO and 2 (1·9%) of the 104 influenza B-infected patients treated with ZN (χ^2^ test, *P* = 0·003) (Table 3C). Although the difference was not statistically significant by the χ^2^ test, biphasic fever was more frequently observed in lower age groups: 13 (7·60%) of 171, 10 (5·88%) of 170, 8 (4·00%) of 200, and 3 (2·70%) of 111 influenza-infected patients ≤7, 8–9, 10–12, and ≥13 years of age, respectively.

**Table 3:** Episodes of biphasic fever in patients with influenza A(H3N2) and B (A), patients with influenza A(H3N2) (B) and patients with influenza B (C) who were treated with zanamivir (ZN) and laninamivir octanoate (LO)

	Cases with biphasic fever (%)	Cases without biphasic fever (%)	Subtotal (%)	Chi square
(A) Influenza A(H3N2) and B				
ZN	6 (1·8)	332 (98·2)	338 (100·0)	*P* < 0·001
LO	28 (8·9)	286 (91·1)	314 (100·0)	
Subtotal	34 (5·2)	618 (94·8)	652 (100·0)	
(B) Influenza A(H3N2)				
ZN	4 (1·7)	230 (98·3)	234 (100·0)	*P* = 0·005
LO	15 (7·0)	198 (93·0)	213 (100·0)	
Subtotal	19 (4·3)	428 (95·7)	447 (100·0)	
(C) Influenza B				
ZN	2 (1·9)	102 (98·1)	104 (100·0)	*P* = 0·003
LO	13 (12·9)	88 (87·1)	101 (100·0)	
Subtotal	15 (7·3)	190 (92·7)	205 (100·0)	

**Figure 4:**
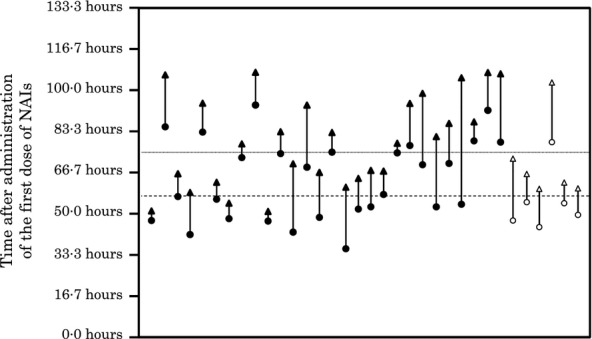
Starting and ending points of biphasic fever after administration of the first dose of the neuraminidase inhibitor (NAI). The starting points of biphasic fever are indicated by white (zanamivir, ZN) and black (laninamivir octanoate) circles, and the ending points of biphasic fever are indicated by white (ZN) and black (laninamivir octanoate) triangles. The starting and ending points of individual patients are bound by a straight line. The starting and ending times of biphasic fever were 36·0–51·0 hours (median: 56·5 hours, broken line) and 94·0–107·0 hours (median: 74·4 hours, dotted line) after administration of the first dose of the NAI, respectively. The duration of biphasic fever was 4·0–51·0 hours (median: 13·5 hours).

The starting and ending times of biphasic fever were 36·0–94·0 hours (median: 56·5 hours) and 51·0–107·0 hours (median: 75·4 hours) after administration of the first dose of the NAI, respectively (Figure 4). The duration of biphasic fever was 4·0–51·0 hours (median: 13·5 hours). Maximum body temperature was 37·6–39·4°C (median: 38·1°C). The differences between the highest temperature in the biphasic phase and the temperature before biphasic fever in individual patients were 0·5–2·8°C (median: 1·5°C).

The logistic regression model showed that the number of biphasic fever episodes increased by 1·19 times for every decrease of 1 year of age (*P* = 0·016) and that the number of biphasic fever episodes in patients treated with LO was 5·80 times greater than that in patients treated with ZN (*P* < 0·001) (Table 4). The logistic regression model for factors influencing episodes of biphasic fever showed no statistically significant differences in sex (*P* = 0·616), types of influenza (*P* = 0·175), vaccination (*P* = 0·726), and time from onset to inhalation (*P* = 0·418) (Table 4).

**Table 4:** Results of logistic regression model to determine factors influencing episodes of biphasic fever

Independent factors	Odds ratio (95% Confidence interval)	*p* value
Age*	1·19 (1·03 to 1·39)	0·016
Sex	1·20 (0·59 to 2·46)	0·616
Anti-influenza drugs**	5·80 (2·51 to 15·79)	<0·001
Types of influenza	1·65 (0·79 to 3·38)	0·175
Vaccination	1·14 (0·55 to 2·34)	0·726
Time from onset to inhalation	0·99 (0·95 to 1·02)	0·418

* The number of biphasic fever episodes increased by 1·19 times for every decrease of 1 year of age.

** The number of biphasic fever episodes in patients treated with laninamivir octanoate was 5·80-times greater than that in patients treated with zanamivir.

## Discussion

The clinical effectiveness of ZN and LO in patients infected with influenza A(H3N2) (*n* = 447) and B (*n* = 205) viruses was evaluated by comparing durations of fever after administration of the first dose of the NAI. In this study, only the duration of fever was used as an index, and other markers of clinical effectiveness (duration of cough, runny nose and sore throat, time to return to normal activities, etc.) were not used.

The duration of fever after administration of the first dose of the NAI showed no statistically significant difference between patients administered ZN and those administered LO (Figure 1A, Table 2). Even when the patients were subdivided into influenza A(H3N2)- and B-infected patients, a significant difference between patients administered ZN and those administered LO was not found in either the influenza A(H3N2)-infected group (log-rank test: χ^2^ = 0·364, d.f. = 1, *P* = 0·546) or influenza B-infected group (log-rank test: χ^2^ = 2·270, d.f. = 1, *P* = 0·102) (figure not shown). In previous studies, no significant difference was observed in time to fever alleviation between patients treated with ZN and those treated with LO,^9^,^10^ being consistent with our results. The duration of fever after administration of the first dose of the NAI was only slightly affected by the time from onset to administration (Table 2). The same result has been obtained in patients with influenza A(H3N2) and influenza B who were treated with OT.^14^

The duration of fever after administration of the first dose of the NAI (ZN or LO) was longer in patients with influenza B infection than in those with influenza A(H3N2) infection (Figure 2, Table 2). This had not been reported before, whereas it has been reported that OT was less effective against influenza B virus infection than against influenza A virus infection^15^,^16^ and that ZN was less effective against influenza B virus infection than against influenza A(H3N2) virus infection.^17^ The 50% inhibitory concentration (IC_50_) of OT for influenza B virus was high (approximately 15 nM), and it has been postulated that this is responsible for the reduced clinical effectiveness of OT against influenza B.^18^ However, all other IC_50_ values of LO and ZN for either influenza A(H3N2) or B strains are relatively low (<4 nM).^18^,^19^ Therefore, except for the reduced clinical effectiveness of OT against influenza B virus, the difference in clinical effectiveness of LO or ZN cannot be explained by *in vitro* IC_50_ values. Contrary to our results, LO has been considered to have the same clinical effectiveness against influenza A(H3N2) and B virus infections.^7^ Because the IC_50_s of LO against influenza B viruses isolated from patients in 2008–2009 and 2010–2011 seasons were higher (19·43 ± 3·58 and 22·13 ± 6·85) than those against influenza A(H3N2) viruses (2·29 ± 0·58 and 3·45 ± 1·34) (data available from the site of Daiichi Sankyo Co., Tokyo, Japan), further clinical studies are needed to evaluate the clinical effectiveness of LO against influenza B virus infection.

The duration of fever after administration of the first dose of the NAI (ZN or LO) in both influenza A(H3N2) and B infections was shorter in older patients (Figure 3, Table 2). It has been reported that the duration of fever was significantly longer for influenza A and B-infected patients aged 0–6 years than for those aged 7 years or over when they were treated with amantadine and OT, respectively,^15^ and that influenza A or B-infected patients aged <6 years exhibited prolonged duration of fever when they were treated with OT.^20^ It has also been reported that a 1-year increment in age of patients infected with influenza A or B virus shortened the fever period by 2·4 hours when they were treated with OT.^21^ Taken together, the results indicate that duration of fever in patients with influenza tends to be longer in young children than older children when they have been treated with OT. Our study showed that the same was true when patients were treated with ZN or LO. These results might be explained partially by immaturity of the immune system against influenza viruses in younger children.^22^

Biphasic fever in influenza has been observed in patients with influenza A(H3N2), A(H1N1), and B virus infections who were not administered NAIs^23^ and in patients with influenza A(H3N2) and B virus infections who were administered OT.^21^ In this study, biphasic fever was observed in 4·3% of the influenza A(H3N2)-infected patients and in 7·3% of the influenza B-infected patients (Table 3). The number of biphasic fever episodes increased by 1·19 times for every decrease of 1 year of age, and biphasic fever episodes were more frequently (5·80 times higher frequency) observed in patients treated with LO than in those treated with ZN (Table 4). Biphasic fever occurred between 36·0 and 107·0 hours after administration of the first dose of the NAI (Figure 4). When the patients who had biphasic fever were removed from the ZN- and LO-treated groups in Figure 1(A), the differences between the two curves ranging from 50 to 110 hours on the horizontal axis in Kaplan–Meier curves disappeared (Figure 1B). Several possibilities can be considered for the difference in rates of biphasic fever between the ZN- and LO-treated groups. One possible explanation is compliance with NAIs. Incomplete inhalation of the single dose of LO, especially in young children, could explain the increase in frequency of biphasic fever in the LO-treated group. On the other hand, ZN is inhaled 10 times more frequently than LO, and children therefore became accustomed to inhalation of ZN. In our study, 37 (10·9%) of the 338 ZN-administered patients actually inhaled ZN < 10 times because of early reduction in fever, and none of those patients had biphasic fever after discontinuance of ZN, indicating its superior effectiveness for preventing biphasic fever. It has been reported that the frequency of biphasic fever in influenza A-infected children in the age group of 1–5 years who were treated with OT was higher than that in children in the age group of 6–12 years who were treated with OT.^21^ Because OT compliance for children in the age group of 1–5 years was considered to be almost the same as that for children in the age group of 6–12 years, other factors (such as immaturity of the immune system against influenza viruses or little chance of prior exposure to influenza) might be associated with biphasic fever. For a similar reason, poor LO compliance alone could not explain the high frequency of biphasic fever in LO-treated patients in our study.

Limitations of this study should be recognized. This study was an observational, not a randomized, study. The decision on whether to administer ZN or LO was left to the discretion of the physician, introducing unmeasured selection bias. As a result, viral loads might be different in the ZO and LO groups. One limitation is the lack of a ‘no treatment’/placebo arm of the study to demonstrate that either ZN or LO shows effectiveness compared to no drug. Another limitation is the lack of virological follow-up, especially in patients who had biphasic fever. It should also be noted that there is a possibility of variability in measurement of body temperature, especially in cases with a short phase of biphasic fever and that information about usage of antipyretics might be helpful for precise analysis of biphasic fever.

In conclusion, our study suggests that episodes of biphasic fever are more frequent in young children and in children treated with LO than in children treated with ZN, although the duration of fever in children treated with LO is the same as that in children treated with ZN.

## Addendum

Keisuke Morita, Akira Inagawa, Akiko Okamura, Shigeru Yamazaki, Satoru Shida, Shinobu Teramoto, Masanori Nakanishi, Mikio Yoshioka, Norihiro Ueno, Mutsuko Konno, Nobuaki Kawamura, Akihito Ishizaka, Kimihiko Takada, Takeyasu Takebayashi, Kazuhiro Tomizawa, Keiji Tsubakihara, Susumu Iizuka, Naoko Nagano, Mutsuo Shibata, Hideto Furuyama, Yoshinori Ogasawara, Yoshihiro Matsuzono, Akemi Koike, Yasutsugu Koga, Mari Murashita, Yoshio Hatae, Hideki Arioka, Susumu Ukae, Tatsuru Yamanaka, and Tohru Watanabe contributed to data collection and analysis.
